# Clinical implication of the serum galectin-1 expression in epithelial ovarian cancer patients

**DOI:** 10.1186/s13048-015-0206-7

**Published:** 2015-11-21

**Authors:** Le Chen, Ying Yao, Lijuan Sun, Jiajia Zhou, Jingshi Liu, Jing Wang, Junjun Li, Jie Tang

**Affiliations:** Department of Gynecology, Hunan Cancer Hospital, the Affiliated Cancer Hospital of Xiangya School of Medicine, Central South University, Changsha, P. R. China; Hunan Cancer Hospital, the Affiliated Cancer Hospital of Xiangya School of Medicine, Central South University, Changsha, P. R. China; Department of Gynecologic oncology, Hunan Cancer Hospital, the Affiliated Cancer Hospital of Xiangya School of Medicine, Central South University, Changsha, P. R. China; Department of Pathology, Hunan Cancer Hospital, the Affiliated Cancer Hospital of Xiangya School of Medicine, Central South University, Changsha, P. R. China

**Keywords:** galectin-1, epithelial ovarian cancer, bio-marker, progression, serum

## Abstract

**Background:**

galectin-1 has been implicated in tumor invasion and metastasis and is frequently over-expressed in epithelial ovarian cancer (EOC), but its potential as a biomarker remains unclear. In this novel study, we have explored the possible use of galectin-1 as a biomarker for EOC.

**Methods:**

galectin-1 in sera was evaluated by ELISA in a pilot panel of EOC patients, healthy volunteers, patients with benign gynecologic tumors or other gynecologic malignancies. We examined galectin-1 expression in EOC tumor samples by Western Blot, qRT-PCR and immunohistochemistry. In vitro experiments were conducted to elucidate the biologic role of galectin-1 in EOC progression using over-expression of galectin-1 in OVCAR-3 cells. We also looked for the association of galectin-1 expression with clinic pathological variables and survival outcomes in EOC.

**Results:**

A significant difference was detected in serum galectin-1 between EOC patients with non-metastatic and those with metastatic disease, but not between EOC patients and healthy volunteers. It increased in recurrent cases and decreased after debulking surgery. Both of galectin-1 mRNA and protein levels were increased in 90 % of the examined EOC tissue samples, compared with a wedge resection of a normal ovary. High galectin-1 in peritumor stroma was primarily detected in advanced stages of EOC. Over expression of galectin-1 significantly increased the ability of OVCAR-3 cells’ migration and invasion.

**Conclusions:**

Our results suggest that galectin-1 might play a role in tumor progression and be associated with poor outcome in EOC. It could be a novel prognostic and progression biomarker in EOC patients.

## Background

Ovarian cancer is the most common gynecological cancer and the leading cause of death from gynecological malignancies in more developed areas, and the second in less developed areas [1]. Approximately, 90 % of ovarian cancer is histological classified as epithelial ovarian cancer. The 5-year survival rate of most EOC patients is only 20 to 30 % with the standard treatment of debulking surgery followed by paclitaxel and platinum (TP) based chemotherapy [1, 2]. The most clinically useful ovarian cancer biomarker, CA125, has been used to assess response to treatment and monitor recurrence of EOC [3], but unfortunately lacks both sensitivity and specificity [4]. A significant proportion of epithelial ovarian cancers do not express CA125, especially tumors of clear cell, undifferentiated, and mucinous histological subtypes [5]. In addition, multiple gynecological conditions can lead to elevated CA125 levels, reducing its overall specificity [6, 7]. There has therefore been a significant interest in the identification and development of new ovarian cancer markers and novel molecular approaches.

Galectins constitute a gene family of widely distributed carbohydrate-binding proteins characterized by their affinity for β-galactoside-containing glycans. Currently there are 14 members in this family [8, 9]. galectin-1, a 14-kDa laminin-binding galectin, a member of the galectin family of β-galactoside-binding proteins classified as a proto-type galectin. It is a homodimer subunit possessing two β-galactoside-binding sites [9, 10]. galectin-1 acts via both intracellular sugar-independent interactions with other proteins, and extracellular sugar-dependent autocrine or paracrine interactions with β-galactoside-containing glycoconjugates [11]. Studies from several groups suggest that galectin-1 may have a role in a variety of physiological and pathological processes including cell-cell and cell-matrix interactions, cell growth, inflammatory reaction and immune regulation [11–13]. Of most relevance to the present study are experimental observations to link the dysregulation of galectin-1 expression to the invasion and metastasis formation of cancer cells [14, 15], promote tumor angiogenesis [12, 16] and protect tumors from host immune responses [17, 18]. In most cases, galectin-1 is up-regulated in cancer cells as reported in thyroid carcinoma by Chiariotti [19] and by Xu [20]. Similarly, increased galectin-1 expression has been correlated with the metastatic potential of several tumorigenic cells, possibly by affecting cell motility and invasion of extracellular matrices [15, 21–23]. It is also detected in neighboring cancer associated fibroblasts [CAFs express α-smooth muscle actin (α-SMA), which can be as a CAF marker] and cancer stroma as reported in the primary prostate cancer by Van den Brule and Berberat PO [24, 25]. Moreover, galectin-1 accumulation in the peritumoral stroma of breast cancer and ovarian cancer regulates both cancer cell proliferation and invasiveness [26, 27]. Recently, it has been shown that galectin-1 is strongly expressed in ovarian cancer and promotes progression and chemoresistance to cisplatin in epithelial ovarian cancer [28, 29]. But its potential as prognostic, diagnostic, or detection marker remains unclear.

In this report, we showed that galectin-1 was able to be released from ovarian cancer cells and cancer associated fibroblasts. The released galectin-1 can be detected in the conditioned culture media of EOC and CAF cells expressing galectin-1, as well as in the peripheral circulation of a majority of EOC patients. Because the expression levels of mRNA and protein galectin-1 were increased in ovary carcinoma samples, compared with normal ovary tissue by western blot and qRT-PCR, the clinic pathologic significance of galectin-1 was further evaluated using IHC of paraffin-embedded archival tissue specimens and statistical analysis. In vitro experiments were performed to determine the function of galectin-1 in cell survival, proliferation, and invasion and migration using over expression of galectin-1 in OVCAR-3 cells. Our findings provide substantial evidence that galectin-1 is a potential biomarker for the prognosis and progression of EOC, but not a screening tool, and their dysregulation might play an important role in the progression of EOC.

## Methods

### Patients

The patients and healthy individuals signed an informed consent form for the study which was reviewed by the Institutional Review Board. All tissue samples were verified by histopathology. A group of formalin-fixed and paraffin embedded archival tissue specimens including 110 cases of primary EOC and 50 cases of normal ovary tissue were used for IHC. Patients were identified from a database containing clinic pathological and follow-up data of all EOC treated with primary debulking surgery followed by TP based chemotherapy according to standard treatment protocols by gynecological oncologists in the Hunan Cancer Hospital (Changsha, China) between 2008 and 2012. Surgical evaluation was used to determine the clinical stage and presence of metastases, whereas histo-pathologic analysis was performed by gynecologic pathologists to assess cancer type and grade. The clinic-pathologic features of the patients used in the present study are shown in Table 3. Seventy patients were serous carcinoma, 25 were mucinous carcinoma, ten were endometriod carcinoma, and five were clear cell carcinoma. All patients were staged according to the 2014 FIGO classification. Follow-up of all patients was performed regularly up to 5 years with gradually increasing intervals. Fifty patients developed tumor recurrence during the postoperative follow-up periods. Among them 40 patients developed pelvic or abdominal metastasis. Ten patients developed pulmonary metastasis, and 20 patients developed lymphatic metastasis. Twenty of them died of the metastatic disease.

### Sera

Blood samples were obtained from patients being seen in the Hunan Cancer Hospital. A total of 140 patients with histological proven diagnosis of primary EOC were included in this study. Samples were allowed to clot, and the serum was stored at −80 °C until assayed. In ten patients, galectin-1 serum levels were measured before and after resection of the primary tumor. Samples were also obtained from 70 normal female blood donors and 40 patients with benign gynecologic tumors from Hunan Province in China (median age of 45 years; range: 21–65 years).

### Tissue samples and construction of tissue microarrays (TMA)

Briefly, primary tumor samples were arrayed using a tissue microarrayer (Beecher Instruments, Silver Spring, Maryland) by taking four representative cores with a diameter of 0.6 mm from marked tumor sites from the individual paraffin embedded tumor block onto a recipient paraffin block at pre-defined array locations. For staining, 4 μm sections were cut from each TMA block and applied to APES-coated slides. H&E staining was performed to verify the presence of tumor in the arrayed samples.

### Immunohistochemical staining (IHC)

TMA sections were dewaxed and rehydrated, whereupon antigen retrieval was performed in a citrate buffer (pH 6.0). Endogenous peroxidase activity was blocked by a 3 % H_2_O_2_ solution, after which sections were incubated overnight at 4 °C with the primary antibody dilution: anti-galectin-1 (Santa Cruz, CA, USA). Sections were subsequently incubated with DAKO Envision + (Dako, Heverlee, Belgium). Antigen-antibody reactions were visualized with 3, 3-diaminobenzidine, the chromogenic substrate for peroxidase, and hematoxylin was used to counter stain the tissue. To evaluate galectin-1 expression, each case was rated according to a score that added a scale of intensity of staining to the area of staining. At least ten high-power fields were chosen randomly, and >1,000 cells were counted for each section. The intensity of staining was graded on the following scale: 0, no staining; 1+, mild staining; 2+, moderate staining; 3+, intense staining. The area of staining was evaluated as follows: 0, no staining of cells in any microscopic fields; 1+, <30 % of tissue stained positive; 2+, between 30 % and 60 % stained positive; 3+, >60 % stained positive. The minimum score when summed (extension + intensity) was therefore, 0, and the maximum, 6. A combined staining score (extension + intensity) of ≤2 was considered to be a negative staining; a score of three was considered to be a weak staining; whereas a score between 4 and 6 was considered to be a strong staining.

### Human EOC cell lines culture

The human EOC cell lines SKOV3 and OVCAR-3 were purchased from American Type Culture Collection (ATCC, USA). All cell lines were maintained in Dulbecco’s modified Eagle’s medium (DMEM) supplemented with 10 % fetal bovine serum and antibiotics in a humidified atmosphere of 95 % air and 5 % CO2 at 37 °C.

### Human cancer associated fibroblast (CAF) cells culture

After several washings with sterile phosphate-buffered saline (PBS), a 2–3 cm^2^ piece of tumor tissue from EOC patients was minced into smooth paste with scissors and incubated on an orbital shaker with 10 ml of PBS and 10 ml of 0.25 % trypsin/25 mM EDTA at 37 °C for about 2 h. The solution containing cells in suspension was filtered by a nylon mesh with 40um micron pores and then centrifuged at 1500 r.p.m. for 5 min. The pellet was plated with 20 ml of RPMI 1640, with 20 % FBS and penicillin/treptomycin and incubated at 37 °C and the medium was exchanged after 48 h for the first time and every third day thereafter.

### Production of the vector expressing galectin-1 and transduction of target cells

The galectin-1 sequences were amplified by PCR, confirmed by sequencing, and then inserted into a pIRES2-ZsGreen1 vector to generate pIRES2-ZsGreen1-galectin-1. OVCAR-3 cells were transfected with pIRES2-ZsGreen1-galectin-1 using LipofectamineTM 2000 kit (Invitrogen) to produce polyclonal cells with stable expression of galectin-1 and confirmed by western blotting.

### Immunocytochemical (ICC)

For immunocytochemical, the CAFs were plated on sterilized coverslips in six-well plates and cultured for 24 h. until the cells adhered, the medium was discarded and the slides harvested for ICC staining. The slides were blocked with 3 % H2O2 for 15 min and 10 % normal goat serum for 15 min at room temperature. Then the slides were incubated withα-SMA (Bioworld Technology, Minion, USA) and galectin-1 ((Santa Cruz, CA, USA) primary antibodies at 4 °C overnight. The HRP-conjugated secondary antibody (Tiangen Biotech, Beijing, China) and 3′-diaminobenzidine tetrahydrochloride were used to visualize signal development, and then the sections were counterstained with hematoxylin. All the sections were observed and photographed with an Axioskop 2 microscope (Carl Zeiss, Oberkochen, Germany).

### Preparation of cell lysate and the conditioned culture media

Whole cell lysates were prepared by adding lysis buffer (62.5 mM Tris–HCl (pH 6.8), 10 % glycerol, 2 % SDS) to 80 % confluent cell cultures. Protein concentration was determined using the BCA assay kit (Pierce, Rockford, IL). For the preparation of the conditioned culture media, cells were grown to 80 % confluence and then with FBS free media for 24 h. The conditioned culture media were collected and centrifuged at 1,000 rpm for 10 min to remove cells and debris, and were further concentrated by Centriplus YM-10 (Millipore).

### ELISA assay for galectin-1

A non-extraction GALECTIN-1 enzyme-linked immunosorbent (ELISA) kit from Kangchen Bio-tech (Shanghai, China) was used for determination of galectin-1 level in sera, cell lystae and cell culture media. Samples were concentrated by Centriplus YM-10 (Millipore) and contested in triplicates and repeated if the correlation coefficient between the absorbance and the amount in the standards was less than 0.95. Long-term storage (≤6 months) of frozen serum or 1 week of storage at 4 °C did not significantly alter galectin-1 content. No difference in galectin-1 measurements was observed after two freeze-thaw cycles.

### Quantitative real-time PCR (qRT-PCR)

Total RNA was extracted with the Trizol Kit (Invitrogen, USA) according to the manufacturer’s instructions. QRT-PCR was performed using a SYBR® Green Realtime PCR Master Mix kit (TOYOBO, Japan) as the manufacturer’s instructions. The primer sequences used were as follows: galectin-1 forward primer 5′-CCGGGGGCCCATCTCTCTCG-3′, reverse primer 5′–CTGGCGAC CAGACCACAAGCC-3′. GAPDH forward primer 5′-TGCACCACCAACTGCTTAGC-3′, reverse primer 5′- GGCATGGACTGTGGTCATGAG −3′. All experiments were repeated at least three times.

### Co-immunoprecipitation and western blotting

The supernatant from whole cell lysates was harvested and immunoprecipitated using the primary antibodies or non-specific IgG as a negative control, which was pre-cleared by protein G magnetic beads at 4 °C overnight. Proteins were separated on 10 or 12 % SDS-PAGE gels and transferred onto nitrocellulose membranes (Bio-Rad, Hercules, CA, USA). About 50 μg of protein extracts of ovary tissues were run on one-dimensional gel and the separated proteins were electro transferred onto nitrocellulose paper and incubated with blocking buffer. Individual membranes were washed and incubated with anti-galectin-1 mouse monoclonal antibody (Santa Cruz, CA, USA), anti-CA125 mouse monoclonal antibody (Santa Cruz, CA, USA) or mouse anti-β-actin antibody (Kangchen Bio-tech Shanghai, China) overnight at room temperature. After the following secondary antibody incubation, the membranes were visualized with chromogens of BCIP (5-bromo-4-chloro −3-indolyl phosphate) and NBT (nitro-bluetetrazoline). All experiments were repeated at least three times.

### In vitro cell growth, proliferation assay, apoptosis assays

The effect of the test agents on cell viability was assessed with the MTT assay. Apoptosis was assessed using an Annexin V-coupled fluorescein isothiocyanate (FITC) apoptosis detection kit (BD Pharmingen, San Diego, CA, USA). Briefly, OVCAR-3/ pIRES2-ZsGreen1 -galectin-1 and OVCAR-3/ pIRES2-ZsGreen1 control cells were removed from a six-well plate by incubation with trypsin-EDTA, washed twice in PBS, and re-suspended in 1 ml of Annexin V-binding buffer at 10^6^cells/ml. Annexin V-coupled FITC and propidium iodide were added (each at 5 μl per 10^5^ cells). Samples were mixed gently, incubated for 15 min at room temperature in the dark, and then subjected to flow cytometry to evaluate the number of apoptotic cells. All experiments were repeated at least three times.

### Transwell migration and invasion assay

In vitro invasion assay was performed in a transwell system (Corning, Corning, USA). Briefly, Matrigel was added to the upper surface of a polycarbonic membrane (pore size 8 μm) to form a thin gel layer, which served as the ECM. The filter was dried in a laminar hood overnight, and then reconstituted with 100 μl of PBS at 37 °C for 2 h. The upper compartment of the filter contained the treated cells at a density of 2 × 10^5^ cells/well in 100 μl of SFM. The bottom filter was filled with 600 μl of conditional medium . After 24 h incubation at 37 °C with 5 % CO_2_, the polycarbonic membrane was fixed with 100 % methanol for 10 min and stained with 0.2 % crystal violet solution, then the cells on the upper surface were completely removed by wiping with a cotton swab. Cells that had penetrated to the lower surface of the filter were counted under an Olympus microscope in three randomized fields at a magnification of 200×. Cell migration assay was carried out in a transwell filter on membrane filters not coated with Matrigel. Migration of cells transduced with pIRES2-ZsGreen1-galectin-1 or pIRES2- ZsGreen1 -control was measured as described in the invasion assay. Each assay was performed at least three times.

### Statistical analyses

All quantitative data are presented as the mean ± SD. SPSS version13.0 was used for statistical analysis. Methods to analyze the statistical significance of differences were student’s two-tailed *t*-test in two groups and one-way ANOVA in multiple groups. Pearson’s chi-square test and Fisher’s exact test were used to assess the statistical significance of the association between galectin-1 expression and clinic pathologic parameters. *P* < 0.05 was considered statistically significant.

## Results

### Serum galectin-1 from normal controls and patients with EOC cancer

**Fig. 1:**
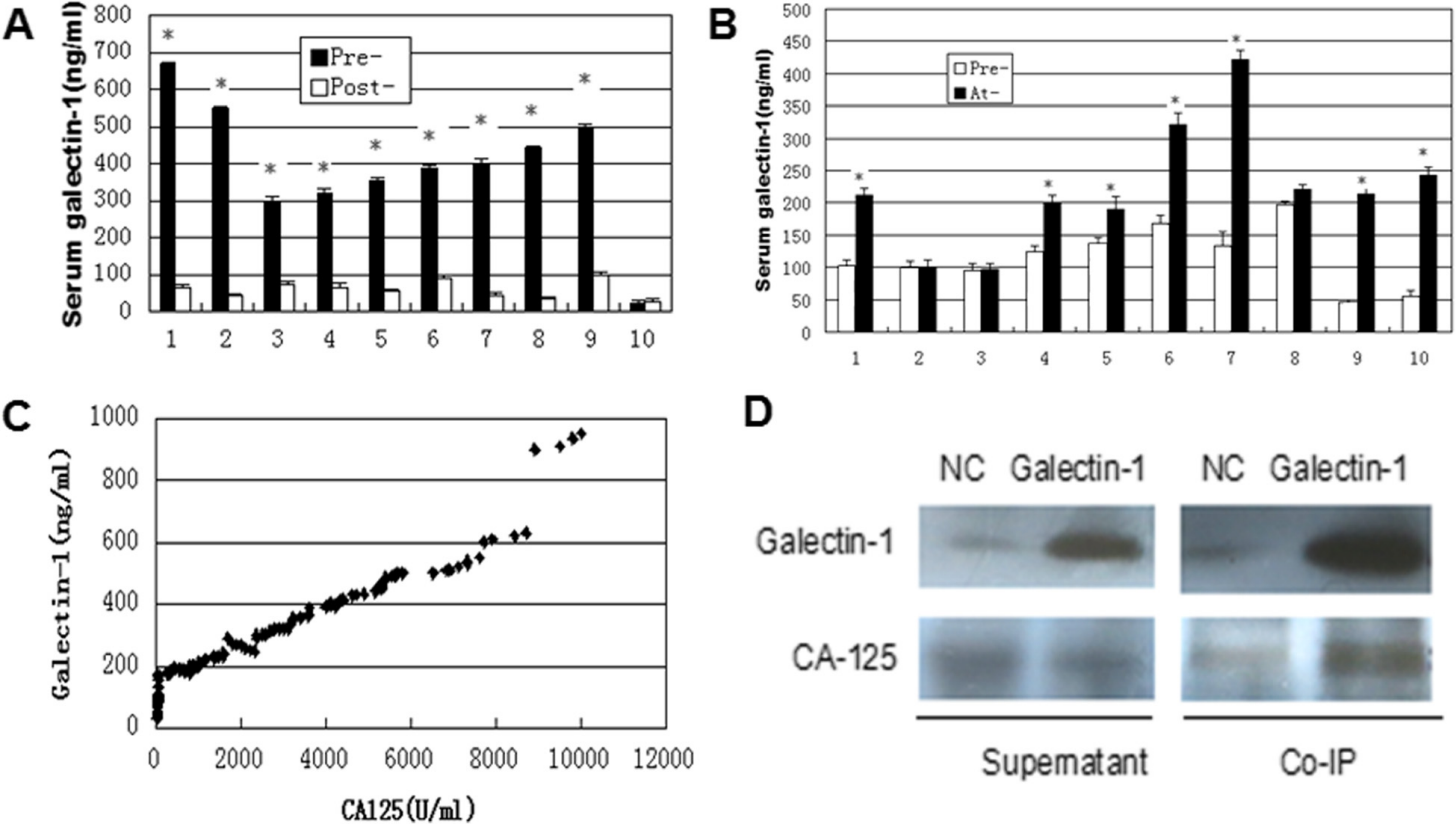
The changes of galectin-1 levels and its relationship with CA125 in the plasma of EOC patients. **a** Serum galectin-1 concentrations in 10 patients with EOC before (Pre-) and 2 days after (Post-) primary debulking surgery. **b** Serum galectin-1 levels before (Pre) and at recurrence (At-) in 10 EOC patients. **c** CA125 levels in plasma of 140 EOC patients are compared to galectin-1 expression levels. **d** Co-immunoprecipitation of CA125 and galectin-1 in galectin-1- pIRES2 - ZsGreen1 transfected OVCAR-3 cells. EOC: epithelial ovarian cancer; NC: negative control; Co-IP: co-immunoprecipitation. *P < 0.05

The levels of galectin-1 in sera of 70 healthy controls varied between 30 and 231 ng/ml (median, 88 ng/ml; 95th percentile, 174.3 ng/ml; Table 1). The 95th percentile of galectin-1 levels (174.3 ng/ml) was arbitrarily taken as the upper limit of normal. There was no significant difference between serum galectin-1 levels and age, menopausal status or blood type. Serum galectin-1 concentrations of healthy individuals and patients with EOC are summarized in Table 1. Five (11 %) of the 45 patients with local disease (stageI) and 47 (50 %) of the 95 patients with metastatic EOC (stageII-IV) showed serum galectin-1 levels above the upper limit of normal. As a whole, there was no a significant difference between healthy individuals, benign gynecologic tumor patients and patients with EOC. There was no a significant difference between different types of ovarian tumor histologies. However, a significant difference in serum galectin-1 was seen between EOC patients with non-metastatic and those with metastatic disease (*P* = 0.034). Furthermore, a significant difference in serum galectin-1 was also observed between EOC patients with different FIGO stage (*P* = 0.041). In 140 patients with early-stage or late-stage EOC, the median galectin-1 concentration in serum was 139 ng/ml (range, 30–950 ng/ml). Serum galectin-1 levels in 10 EOC patients were measured before and 2 days after primary debulking surgery. In nine of these cases, there was a decrease in the galectin-1 serum concentrations after surgery (Fig. 1a). One patient with pelvic lymph node involvement had a preoperative galectin-1 serum level of 595 ng/ml that dropped to165 ng/ml within 2 days after satisfied surgery. In contrast, in one patient with localized tumor with normal galectin-1 serum level before surgery, no significant change was observed in galectin-1 serum concentration. Interestingly, serum concentrations of galectin-1 increased in 7/10 recurrent cases compared with the level at the end of treatment (Fig. 1b).

**Table 1:** Levels of galectin-1 in the sera of EOC patients and healthy controls

		galectin-1 (ng/ml)				
Diagnosis	No. of patients	Median	5th percentile	95th percentile	Range	P
Healthy donors	70	88	30	174.3	30–231	0.15^a^
Benign Gyn Tumor	60	78	32.5	169.8	30–229	0.38^b^
Other Gyn						
Malignancies	50	92	33.5	176.5	30–240	0.042^c^
Histologies						
Serous	105	191	31.5	760		
Mucinous	45	202	34.5	812		
Endometroid	37	179	29.8	745		
Clear cell	23	182	30.2	789		0.784^d^
EOC	140	149	34.5	828	30–950	
Nonmetastatic	45	69	36.2	169.3	30–163	
Metastatic	95	370	40	840	30–950	0.034^e^
EOC stage						
I	44	69	36.2	169.3	30–163	
II	35	260	39	370	30–390	
III	46	352	180	590	170–610	
IV	15	630	505	890	480–950	0.041^f^

*a*-*f* Ps refers to the difference between the cohorts of healthy individuals and EOC patients ^a^, or the difference of EOC patients and patients with benign gynecologic tumors ^b^, or the difference of EOC patients and patients with other gynecologic malignancies ^c^, or between the groups of EOC patients with different histology ^d^, or the difference between the groups of patients with nonmetastatic and metastatic disease ^e^ or the groups of patients with stageI- IV^f^. *EOC* epithelial ovarian cancer, *Gyn* gynecologic

### Comparison of galectin-1 and CA125 levels

CA125 is the most clinically useful ovarian cancer biomarker. We therefore wished to compare the galectin-1 positivity with CA125 values in our tumor panel. EOC patients that demonstrated high levels of CA125 also tended to exhibit high levels of galectin-1 (Fig. 1c). At a CA125 threshold of 35 U/ml, fifteen of CA125-negative plasma samples were in galectin-1-positive range. 98 of the 140 cancer patients identified as positive by CA125 were also positive using galectin-1 and 22 patients were negative for both (Table 2). Using Fisher’s exact test, the association between galectin-1 and CA125 positivity was shown to be highly significant (*P* = 0.001), confirming the lack of independence between these two markers. A false positive obtained with CA125 was negative obtained by galectin-1. Interestingly, using co-immunoprecipitation we found that the protein of galectin-1 combined with CA125 in galectin-1- pIRES2-ZsGreen1 transfected OVCAR-3 cells (Fig. 1d). It has been also implicated that CA125 represents a novel counter receptor for galectin-1 in Hela cells [30].

**Table 2:** CA125 and galectin-1 in EOC plasma samples

	galectin-1^+^	galectin-1^−^
CA125 positive	98	5
CA125 negative	15	22

*EOC* epithelial ovarian cancer

### Cancer cells and cancer associated fibroblasts (CAF) in culture release galectin-1 proteins

**Fig. 2:**
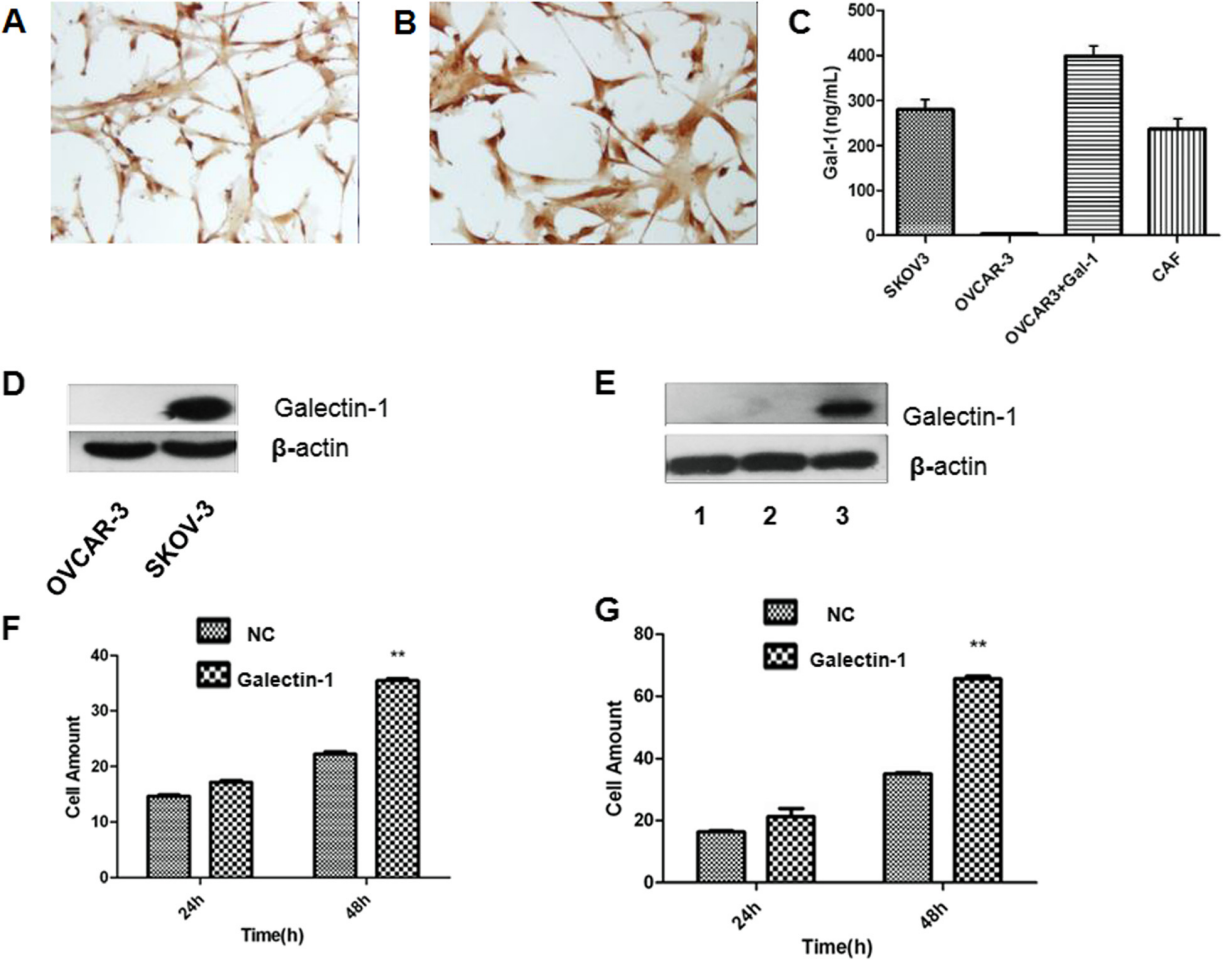
Expression of galectin-1 in various EOC cell lines and in vitro invasion and migration assays using pIRES2-ZsGreen1-galectin-1 transduced OVCAR-3 cells. **a**–**b** The basal expression of galectin-1 (**a**, ×40) determined by ICC in CAF cells confirmed by α-SMA staining (**b**, ×40). **c** ELISA detected galectin-1 in conditioned culture media of SKOV3 cells, OVCAR-3 cells, galectin-1- pIRES2 - ZsGreen1 transfected OVCAR-3 cells and CAF cells. **d** Basal expression of galectin-1 in ovarian cancer cell lines. **e** The expression of galectin-1 was increased by transfecting OVCAR-3 cells with pIRES2-ZsGreen1-galectin-1. 1 represents OVCAR-3 alone; 2 represents pIRES2-ZsGreen1 transfected OVCAR-3 cells; 3 represents pIRES2-ZsGreen1-galectin-1 transfected OVCAR-3 cells. **f**–**g** Up-regulation of galectin-1 by pIRES2-ZsGreen1-galectin-1 increased the capacity of invasion (**f**) and migration (**g**) for OVCAR-3 cells. NC: Negative control; EOC: epithelial ovarian cancer; galectin-1:galectin-1; CAF: cancer associated fibroblast; IHC: Immunohistochemistry. **P < 0.01

In order to test the possibility that galectin-1 may be released from cancer cells and CAF, we looked for the presence of galectin-1 in conditioned culture media from EOC cell lines and CAF cells. A primary cell line of CAF expressing galectin-1 (Fig. 2a) was cultured from an EOC patient and confirmed by ICC using anti-α-SMA (Fig. 2b). Conditioned culture media from OVCAR-3, SKOV3 and CAF cells were collected and concentrated. ELISA was performed to detect galectin-1 in the conditioned culture media (Fig. 2c). Interestingly, the presence of galectin-1 in the media corresponded to expression of galectin-1 in the various cell lines. galectin-1 was not detected in the media of OVCAR-3 lacking galectin-1 expression. However, glectin-1- pIRES2-ZsGreen1 transfected OVCAR-3 cells showed high levels of galectin-1 both in the media and the lysate. Overall, our findings show that, when expressed in a cell, a fraction of the galectin-1 protein can be released in the media. Both EOC cells and CAF cells are able to release galectin-1 in the media.

### galectin-1 is involved in EOC cell migration and invasion in vitro

To elucidate the role of galectin-1 in EOC progression, pIRES2-ZsGreen1 vector was used to increase galectin-1 expression in OVCAR-3 cells which have no galectin-1 protein expression (Fig. 2d). Up-regulation of galectin-1 expression was observed in galectin-1-pIRES2-ZsGreen1 transfected OVCAR-3 cells (relative to the GFP control) (Fig. 2e). galectin-1 up-regulation significantly increased migration and invasion abilities of OVCAR-3 cells compared with the GFP control (*P* < 0.01) (Fig. 2f–g). However, no effect was observed in proliferation and apoptosis for OVCAR-3 cells (data not showed).

### Identification of high galectin-1 expression in EOC tumor specimens and statistical analysis

**Fig. 3:**
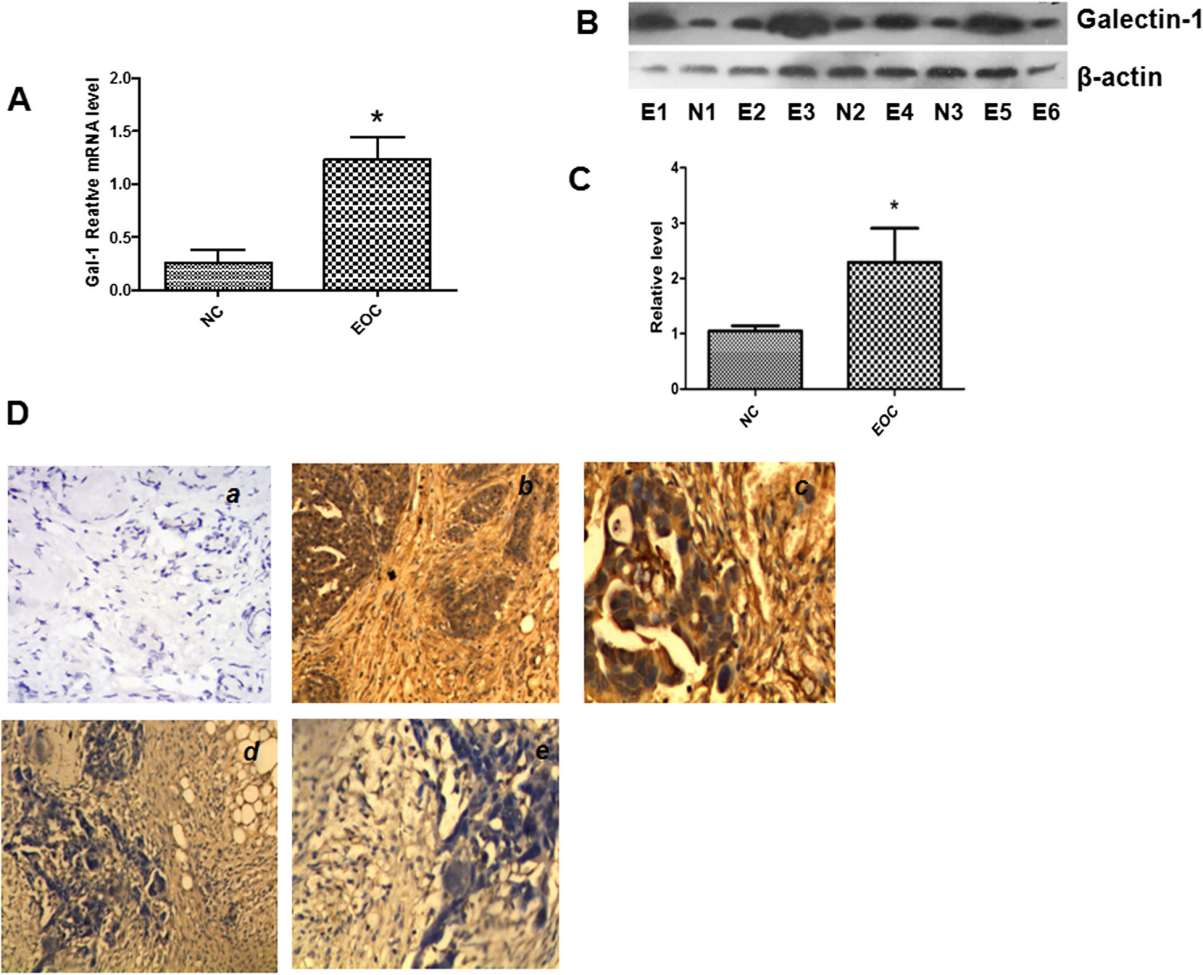
Representative results of galectin-1 expression in the tissue specimens. **a** QRT-PCR shows changes in the expression levels of galectin-1 in EOC cancerous tissue and normal ovary sample. **b** Western blotting shows changes in the expression levels of galectin-1 in EOC cancerous tissue and normal ovary samples. **c** Histogram of relative changes in the expression level of galectin-1 protein in the tumor and normal tissues as determined by densitometric analysis. **d** The typical examples of galectin-1 expression in human EOC determined by immunohistochemistry: (*a*) Negative galectin-1 expression in normal ovary tissue (×200). **b**, **c** Strong galectin-1 expression in EOC cells and cancer-associated stroma [×200 for (*b*) and ×400 for (*c*)]. **d**, **e** Negative galectin-1 expression in cancer cells but strong galectin-1 expression in cancer associated stroma [×200 for (*d*) and ×400 for (*e*)]. *P < 0.05. EOC: epithelial ovarian cancer; galectin-1:galectin-1; E: EOC cancerous tissue; N: normal ovary sample; NC: Negative control

Western blotting and qRT-PCR were done to detect the expression levels of galectin-1 in cancerous ovary tissue from 36 EOC patients compared with a wedge resection of 32 normal ovaries. As shown in Fig. 3a, b and c, galectin-1 was significantly up-regulated in both mRNA and protein levels (*P* < 0.05) in 32 (90 %) of EOC patients. Because of its cell type specificity, IHC was considered as the most reliable detection technique, allowing discrimination between normal and neoplastic tissue. After showing the increased expression of galectin-1 in epithelial ovarian cancerous tissue compared to the normal ovarian tissue by western blot and qRT-PCR analysis, we investigated the level of galectin-1 expression in the pathologic specimen of EOC patients using the IHC method. galectin-1 expression was heterogeneous from sample to sample.

### Levels of galectin-1 expression in cancer cells

galectin-1 was stained both in cancer cells with a cytoplasmic pattern and in stromal cells (Fig. 3d). The levels of galectin-1 expression in cancer cells were analyzed to the pathologic and clinical information (Table 3). The levels of galectin-1 expression did not show any statistically significant changes according to tumor stage, tumor invasiveness, presence of lymph node metastasis.

**Table 3:** galectin-1 expression in cancer cells and cancer-associated stromal cells along various pathologic characteristics

Tumor pathology	Staining location	Levels of expression		Total	p value
		Weak (0, 3+)	Strong (4+, 6+)		
FIGO stage	*CAS* ^*a*^				
	I	15 (76.0 %)	5 (24.0 %)	20	0.020
	II	12 (43.0 %)	17 (57.0 %)	29	
	III	12 (32.0 %)	25 (68.0 %)	37	
	IV	3 (12.4 %)	21 (87.6 %)	24	
	*EOC* ^*b*^ *cells*				
	I	7 (36.5 %)	13 (63.5 %)	20	0.478
	II	4 (14.4 %)	25 (85.6 %)	29	
	III	8 (21.5 %)	29 (78.5 %)	37	
	IV	8 (35.5 %)	16 (64.5 %)	24	
Lymph	*CAS* ^*a*^				
node metastasis	Metastasis (−)	32 (48.2 %)	35 (51.8 %)	67	0.041
	Metastasis (+)	12 (28.5 %)	31 (71.5 %)	43	
	*EOC* ^*b*^ *cells*				
	Metastasis (−)	14 (21.6 %)	53 (78.4 %)	67	0.214
	Metastasis (+)	14 (32.8 %)	29 (67.2 %)	43	
Recurrence	*CAS* ^*a*^				
	Recurrence (−)	46 (77.3 %)	14 (22.7 %)	60	0.002
	Recurrence (+)	15 (30.8 %)	35 (69.2 %)	50	
	*EOC* ^*b*^ *cells*				
	Recurrence (−)	24 (39.5 %)	36 (60.5 %)	60	0.245
	Recurrence (+)	13 (25.6 %)	37 (74.4 %)	50	

^a^Cancer-associated stromal. ^b^Epithelial ovarian cancer

### Levels of galectin-1 expression in cancer-associated stromal (CAS) cells

Examination of the stroma associated with the carcinoma cells demonstrated that it was positive for galectin-1 immunostaining in a large majority of samples (score 3–6 in 81 of 110 cases). However, the stroma associated with the normal tissue, when present on the slide, was characterized by low or absent immunostaining (score 0–3 in 46 of 50 cases). The levels of galectin-1 expression in CAS cells of epithelial ovarian cancer tissues varied among patients. In contrast to the results of galectin-1 staining in the cancer cell, statistical analysis of the data revealed the staining results of the stromal cells showed significant correlation with pathologic variables of the EOC patients (Table 3). High levels of galectin-1 expression in stromal cells were observed in the invasive carcinoma compared to the non-invasive carcinoma (*p* = 0.004), and the levels of galectin-1 expression in CAS cells showed positive correlation with FIGO stages of EOC. The stromal galectin-1 expression were significantly higher in patients with lymph node metastasis (*p* = 0.041). Interestingly, when EOC patients were divided into two groups according to staining intensity of immunohistochemistry, namely a weak expression group (score = 0–3) and a strong expression group (score = 4–6), the recurrent rate in 3 years was higher in the strong galectin-1 expression group than that in the weak galectin-1 expression group (*P* = 0.002) (Table 3).

## Discussion

There has been a sustained interest in the identification of bio-markers for the prognosis and progression of EOC since quite a few epithelial ovarian cancers are CA125 negative and it is not very specific or sensitive. Additional EOC serum markers will be required to identified for all patients in an initial phase of screening as 20 % of ovarian cancers have little or no expression of CA125 [4]. More than 30 serum markers have been evaluated alone and in combination with CA125 by different investigators, such as Kallikreins, osteopontin, leptin, HE-4, LPA, MUC1 and SLP I [31–35] can improve the predictive value in the past several years through various approaches, including gene expression profiling and proteomics analysis of ovarian tumors. But the usefulness of these markers alone or in various combinations still remains to be determined [36]. galectin-1 has emerged as a protein commonly elevated in ovarian cancer [28, 29]. Here, our study demonstrates that galectin-1 expression is increased in EOC patients, preferentially in sera and in the adjacent peritumoral stroma. We have measured serum galectin-1 concentrations in 140 patients with EOC. Compared with the levels in 70 healthy individuals, only about 37 % of examined EOC cases showed galectin-1 concentration more than the cutoff level; however, the incidence of supernormal levels of galectin-1 was elevated in relation to tumor progression. Serum galectin-1 levels were significantly higher in patients with metastatic disease compared with patients with localized tumors. This tendency, that the increase in serum galectin-1 levels was associated with the occurrence of metastasis. Our results could imply that the metastatic spread of malignant tumors involves a higher level of galectin-1 in the circulation. We are proposing that increasing the serum level of galectin-1 may favor metastasis by the following-mentioned modalities: (a) enhancing the adhesive interactions between tumor cells and the extracellular matrix proteins, such as laminin, fibronectin, and vitronectin [37]; (b) promoting tumor cell embolization through increased cell adhesion and dissemination of tumor cells in the circulation [38, 39]; (c) elevating tumor vascular permeability [40]; and (d) conferring a selective survival advantage to metastatic cells (anoikis) [19, 41, 42]. Alternatively, galectin-1 serum levels may reflect an immune reaction to the tumor load from inflammatory cells that are known to express galectin-1. However, this seems unlikely because we found no correlation between the extent of the inflammatory response in operable EOC and further disease progression. The source of increased serum galectin-1 in cancer patients remains unclear. According to our results, that removal of the tumor decreased serum galectin-1 concentrations, tumor tissues are likely to produce and secrete galectin-1 in sera. However, immunostaining of cancerous tissue showed that galectin-1 was expressed not only on malignant cells but also in stromal cells (mainly fibroblasts) near cancer nests, and the stromal cells immediately adjacent to cancer nests have a higher galectin-1 expression in comparison to those cells farther away from the nests. Both EOC cells and CAF released galectin-1 in the conditioned culture media. These results suggest that circulating galectin-1 is generated not only by tumor cells but also from peritumoral stromal cells.

In addition, we performed both qRT-PCR and western blot to demonstrate high expression of galectin-1 in EOC tumor specimens. The clinic pathologic significance of galectin-1 was further evaluated using immunohistochemistry of paraffin-embedded archival tissue specimens and statistical analysis. The expression pattern of the galectin-1accumulation in the stroma has also been reported surrounding thyroid [19], colon [43, 44], gliomas [22], prostate carcinoma [24] and beast cancer [27]. Our report demonstrates that high galectin-1 expression in cancer-associated stroma correlates with various clinic pathologic parameters, such as tumor invasiveness, advanced stage, metastasis and higher recurrent rate. Van den Bru˄le et al. [24, 26] observed the expression of galectin-1 mRNA in peritumoral fibroblasts close to ovarian carcinoma cells in human ovarian carcinoma tissues, and demonstrated the galectin-1 secretion from the activated fibroblast cell line. Moreover, the conditioned media from certain ovarian cell lines were shown to modulate the galectin-1 expression in fibroblasts regardless of galectin-1 expression status in primary cell lines. Berberat et al. [25] also reported that galectin-1 mRNA expression in stromal fibroblasts by in situ hybridization in pancreatic cancer. Two possible mechanisms may explain this. Firstly, galectin-1-expressing carcinoma cells can synthesize and secrete galectin-1into stromal cells using its non-classical secretory pathway [45]. Secondly, galectin-1 can be synthesized by stromal cells, especially stromal fibroblasts, as they get stimulated by oncologic signals from carcinoma cells or from ECM during ECM remodeling. Therefore, at least in part the galectin-1 expression in cancer-associated stromal tissue is due to the synthesis in stromal fibroblasts, and this stromal galectin-1 expression can be modulated by conditions of tumor microenvironment. Although it is not yet fully elucidated, the role of galectin-1 in cancer-associated stroma is certainly an interesting issue due to the highlighted significance of tumor-stromal interactions [46, 47]. In vitro experiments, we showed that over expression of galectin-1 in OVCAR-3 cells significantly increased the ability of cells’ migration and invasion.

## Conclusions

Our study demonstrates the increased galectin-1 expression in EOC and stresses the clinical significance of galectin-1 expression in sera and cancer-associated stroma, a finding that could be important for cancer progression. When taken together with galectin-1’s role in cancer cells migration and invasion, we believe that galectin-1 can be a key player in tumor stroma interaction in EOC, and the detection of increased galectin-1 levels in the serum of certain patients with cancer may reflect biological aspects of tumor behavior associated with a metastasizing phenotype. More studies are warranted to determine the clinical value of circulating galectin-1 in patients with early-stage cancer as a predictor of tumor invasion and metastasis. The influence of host-derived versus cancer cell–derived galectin-1 on cancer progression remains to be further elucidated. These data support a role for galectin-1 to be a novel prognostic and progression biomarker in EOC patients.

## Abbreviations

EOC: Epithelial ovarian cancer
ELISA: Enzyme-linked immunosorbent assay
IHC: Immunohistochemistry
ICC: Immunocytochemical
CAF: Cancer associated fibroblast
CAS: Cancer associated stroma
